# Leveraging stories of cardiac amyloidosis patients of African ancestry or descent to support patient-derived data elements for efficient diagnosis and treatment

**DOI:** 10.3389/fphar.2023.1276396

**Published:** 2023-11-24

**Authors:** Rachele M. Hendricks-Sturrup, Lauren M. Edgar, Christine Y. Lu

**Affiliations:** ^1^ National Alliance Against Disparities in Patient Health, Woodbridge, VA, United States; ^2^ Department of Population Medicine, Harvard Pilgrim Healthcare Institute and Harvard Medical School, Boston, MA, United States; ^3^ Duke-Margolis Center for Health Policy, Washington, DC, United States; ^4^ Southern Nevada Black Nurses Association, Las Vegas, NV, United States; ^5^ School of Pharmacy, Faculty of Medicine and Health, The University of Sydney, Sydney, NSW, Australia; ^6^ Kolling Institute, Faculty of Medicine and Health, The University of Sydney and the Northern Sydney Local Health District, Sydney, NSW, Australia

**Keywords:** amyloidosis, African, patient-reported outcomes, electronic health record, diagnosis, rare disease, data element

## Introduction

Storytelling is a powerful tool that continuously drives knowledge development and sharing about the patient experience with managing hereditary diseases and comorbidities, including patient values and preferences, and medication adherence and persistence. Patient stories are often key to developing patient-reported outcomes (PROs) data that are specific to or reflective of a patient’s lived experience, most bothersome or frequent symptoms, and socioeconomic circumstances (Kwan et al., 2016; Boyd et al., 2023; Boyd et al., 2023). Thus, there is power in the potential to leverage patient stories to inform the development of new or refinement of existing PRO collection tools for more accurate and timely diagnosis and optimizing the management and treatment of rare diseases, which are often challenging to diagnose particularly among minority populations (e.g., Patient Reported Outcomes Measurement Information System [PROMIS^®^], United States [US] National Cancer Institute’s PRO-CTCAE, Transthyretin Amyloidosis—Quality of Life Questionnaire [ATTR-QOL], interviews, and focus groups; D’Souza et al., 2023; O’Connor, 2023). Below we discuss the potential benefits of incorporating patient stories into PRO instruments to screen and manage African, African American, and/or Afro-Caribbean (A/AA/AC) patients with suspected amyloidosis, a rare disease that occurs when a protein called amyloid builds up in organs (heart, kidneys, liver, spleen, digestive tract, and nervous system).

There are 18 different types of systemic forms of amyloidosis, as well as 22 localized forms. Two major forms of amyloidosis include but are not limited to immunoglobulin light chain (AL) and transthyretin amyloidosis (ATTR; Benson et al., 2020). Additional forms of amyloidosis beyond these two types are secondary, dialysis-related, hereditary (hATTR), organ-specific, insulin-related, or associated with a myriad of pathologies (Gorevic, 2023). Based on stories shared broadly to date, A/AA/AC patients with amyloidosis often experience a lengthy diagnostic odyssey following initial presence of clinical symptoms. Confusion among themselves, their families, and their healthcare providers often cause delays in diagnosis, misdiagnosis, and/or treatment. Such delays directly contribute to often-fatal outcomes observed. Therefore, all of these factors considered, the true prevalence of AL and ATTR amyloidosis, among other forms, across A/AA/AC populations is neither well-understood nor well-documented in the literature.

To help address this issue and better serve these populations and health systems they encounter, we highlight and discuss patient stories from A/AA/AC patients living with AL and ATTR amyloidosis. We also, 1) summarize the underlying disease etiology; 2) share A/AA/AC amyloidosis patient stories to inform or enrich PRO themes that may convey the important spectrum of the patient experience, from symptom onset, to diagnosis, to treatment and/or management; and 3) inform efforts toward the development of data elements, fields, and features within electronic health record systems that may better align with these patient experiences and stories.

## Signs and symptoms of amyloidosis in A/AA/AC patients

**FIGURE 1:**
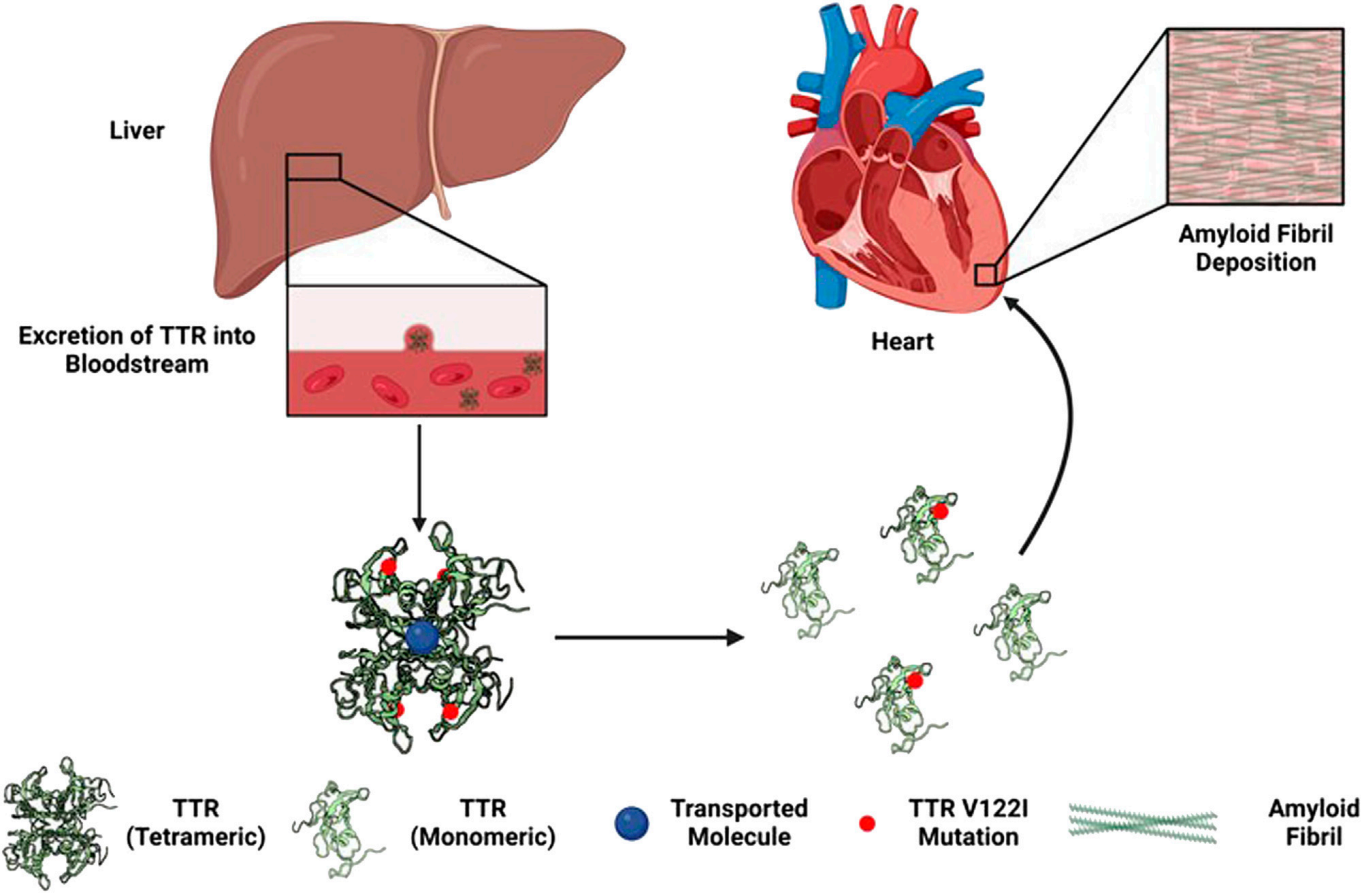
Development of hereditary transthyretin cardiac amyloidosis via *TTR* V122I (pV142I) allele mutation (Williams et al., 2022).

Cardiac amyloidosis is caused by abnormal amyloid protein aggregate deposits that form insoluble plaques in the myocardium, leading to a progressive disorder that often results in restrictive cardiomyopathy (see Figure 1; Ruberg et al., 2019; Williams et al., 2022). Tetrameric thyroxine transport protein transthyretin (*TTR*) is a homotetrameric protein complex that is synthesized in and secreted by the human liver for retinol and vitamin A transfer within the circulatory system (Saelices and Cascio, 2015; Saelices and Johnson, 2015). The most common mutation associated with hATTR is the V122I (pV142I) allele, whereas a valine-to-isoleucine substitution at position 122 (*TTR* V122I; pV142I) in *TTR*-derived fibrils (Buxbaum and Ruberg, 2017). Thus, suspected cases of transthyretin amyloid cardiomyopathy (ATTR-CM) and hATTR among individuals of A/AA/AC descent must often include, in addition to the gold standard cardiac biopsy, molecular testing to confirm the presence or absence of a *TTR* mutation (Dungu, 2015).

hATTR is a hereditary form of cardiac amyloidosis, versus the non-hereditary or wild-type form, that can be fatal, as ATTR accompanied by cardiomyopathy (i.e., ATTR-CM) and heart failure (i.e., fatal arrhythmias or complete heart blockage) are common (Jain and Zahra, 2023). hATTR diagnosis is often delayed, with a late diagnosis often translating into two to 3 years of remaining life expectancy (Jain and Zahra, 2023). Liver or consolidated heart and liver transplantation have been the main treatment for patients with changes caused by ATTR, including those with cardiovascular signs and symptoms. In addition, tafamidis treatment, among others (i.e., patisiran, vutrisiran, and inotersen), may increase the life expectancy of ATTR-CM patients by 4.19 years (Tran et al., 2020). Tafamidis (i.e., Vyndaqel) is presently regulated by the US Food and Drug Administration with clinical pharmacology and clinical study labeling sections, corresponding to *TTR* as its corresponding pharmacogenomic biomarker (Center for Drug Evaluation and Research, 2023).

ATTR-CM is an under-recognized cause of cardiac/heart weakness and failure among middle aged and geriatric adults. Moreover, ATTR-CM disproportionately affects individuals of A/AA/AC descent; in ∼3.4% of populations with ancestral origins from coastal west Africa and clinical signs of hATTR, a high frequency of the *TTR* V122I (pV142I) allele can be observed (Jacobson et al., 2016).

AL amyloidosis is the most common form of amyloidosis, whereas the immune system produces “light chains” or abnormal antibodies/immunoglobulins. In AL amyloidosis, light chains are deposited in major organs, such as the heart and nerves, thereby compromising their optimal function. AL amyloidosis is also associated multiple myeloma, a form of cancer that disproportionately affects A/AA/AC populations, lending AL amyloidosis symptoms as often treated in cancer centers (Kumar et al., 2017; Lee et al., 2021; Gorevic, 2023; *AL (Primary) Amyloidosis*, n.d.). However, the incidence of AL amyloidosis related multiple myeloma in A/AA/AC populations in the US remains unclear.

Because amyloidosis does not present as a single condition when phenotypes are observed among A/AA/AC patients, clinicians may erroneously steer towards clinical reasoning that supports diagnosis and treatment of a neurological disorder or cardiac and musculoskeletal manifestations/disorders (Nativi-Nicolau et al., 2022). In other instances, clinicians may rule signs and symptoms as idiopathic or of unknown cause (Nativi-Nicolau et al., 2022). Therefore, when seeking solutions to diagnose A/AA/AC patients more efficiently and effectively, it is necessary to acknowledge overall that 1) diagnosis can be difficult due to heterogeneity in phenotypes; and 2) clinical signs and clues of AL and ATTR amyloidosis among suspected A/AA/AC patients may differ from more generalized cases, warranting clinical suspicion and/or equipoise (*Geographic Origins, Diagnosis and Treatment of Hereditary Amyloidosis - African Americans*, 2023).

## Leveraging A/AA/AC amyloidosis patient stories for better care

Prior work and A/AA/AC amyloidosis patient stories have highlighted important themes on which to build and that likely capture the range of lived experiences among populations managing genetic diseases and their comorbidities (National Black Nurses Association [NBNA], 2019; Hendricks-Sturrup, 2021; Brown, 2022; *Genetic Origins, Diagnosis and Treatment of Hereditary Amyloidosis on African Americans*, 2022; Baxton II, n.d.; Beckwith, n.d.; Foster, n.d.; Jackson-Webb, n.d.; Strickland, n.d.). These themes are as follows:

• Access to clinical, molecular diagnostic testing for *TTR* mutations, as lack of access to testing may hinder prior/initial authorization for pharmacogenomic treatment for health-compromised patients with hATTR (Blue Cross Blue Shield, 2019; Cigna, 2023; UnitedHealthcare, 2023).
• Diagnosed populations may lose life insurance coverage, especially if molecularly diagnosed (i.e., genetic testing) during late disease stages.
• Access to follow-up testing and/or care (i.e., tissue biopsy, echocardiogram, cardiac magnetic resonance imaging, radionuclide imaging, technetium pyrophosphate scan, etc.) (Blue Cross Blue Shield, 2019; Cigna, 2023; UnitedHealthcare, 2023).
• Lack of personal and family understanding of amyloidosis.
• Struggle to maintain and active lifestyle in later life.
• Clinical signs tend to include a mixture of carpal tunnel syndrome, arrhythmia, gastrointestinal issues, and common signs of heart failure.
• Underdiagnosis of the disease in African American populations results in late-stage diagnosis, contributing to poor outcomes and prognosis due to poor stabilization that is needed to seek and engage in preventive care.
• Chronic, acute, and prolonged stress, including general malaise, affects day-to-day life functioning and increases risk of mental illness (e.g., depression, anxiety, etc.).
• Fragmented, under-resourced, under-educated, and underprepared health systems and healthcare providers contribute to delayed diagnosis.

A/AA/AC nursing professionals, who often spend time at the bedside learning patients’ stories and experiences to document such information within electronic health records, and other health system stakeholders consider patient community stories as powerful resources to directly address health disparities through intentional data collection, use, and reporting (Hendricks-Sturrup, 2021; Hendricks-Sturrup et al., 2021; Evidation, n.d.). Moreover, A/AA/AC nursing professionals, in addition to caregivers of A/AA/AC patients with AL and ATTR amyloidosis, are well-positioned to inform the development of both objective and subjective PROs (i.e., PROMIS^®^, PRO-CTCAE, ATTR-QOL, interviews and focus groups) intended to capture the AL and ATTR A/AA/AC patient experience within the electronic health record. For example, PROMIS^®^, PRO-CTCAE, and ATTR-QOL do not currently contain domains focused on access to molecular testing, access to non-health insurance following testing, follow-up testing and/or care, concern about late-stage diagnosis and poor symptom stabilization, and experiences navigating complex health systems that contribute to delayed diagnosis. Therefore, it is imperative that such stakeholders learn from and disseminate these themes to encourage and support their integration into electronic PROs. Supplementary Table S1 provides two examples reported in recent literature of how PROs are currently used and embedded into electronic health records along with reported evidence of patient management and treatment outcomes.

## Discussion

Consideration these themes across A/AA/AC amyloidosis patient stories is necessary to augment PRO instruments and collection processes that are typically used to understand quality of life, address diagnosis and treatment disparities, and reduce the likelihood of diagnostic odyssey among A/AA/AC patients with amyloidosis. Given that A/AA/AC patients with suspected amyloidosis may lack access to amyloidosis centers of excellence, the present themes herein should inform attempts to identify and properly resource and educate healthcare providers located outside of such institutions where A/AA/AC patient populations are prevalent (Nativi-Nicolau et al., 2021).

The themes above may also inform novel approaches intended to address diagnosis and treatment disparities among A/AA/AC patients with amyloidosis (Alexander et al., 2018; Obi et al., 2022). For instance, A/AA/AC amyloidosis patient stories collected and assessed using advanced computing technologies, such as artificial intelligence (i.e., natural language processing of unstructured patient story data and clinical notes, etc.), coupled with systematically documenting genetic and social determinant of health International Classification of Diseases (ICD)-10 Z codes, could be a promising strategy to address amyloidosis diagnosis and treatment disparities among A/AA/AC patients (Center for Medicare and Medicaid, 2023; Centers for Medicare and Medicaid, 2023). Specifically, leveraging artificial intelligence for the purpose of effectively and efficiently identifying and diagnosing A/AA/AC patients with suspected amyloidosis, based on distinct clinical and diagnostic clues, could be key to addressing health disparities more rapidly and efficiently (*Geographic Origins, Diagnosis and Treatment of Hereditary Amyloidosis - African Americans*, 2023).

A/AA/AC patients with suspected amyloidosis and their families, as well as healthcare providers, health researchers, and policymakers must learn from diagnosed A/AA/AC amyloidosis patient stories to facilitate more informed decision-making. As cardiovascular disease continues to be a leading cause of death overall and among A/AA/AC populations in the United States, targeted and sustained research funding and support to empower racial/ethnic minority patient stories across the data lifecycle should be a national priority to address health disparities (Data Across Sectors for Health [DASH], All In and National Alliance Against Disparities in Patient Health [NADPH], 2022; FastStats, 2023). Similar approaches to improve diagnostic efficiency and accuracy in A/AA/AC amyloidosis patients could be applied to shorten or minimize diagnostic odyssey among patients of other genetically-derived rare diseases, lending to stronger opportunities to provide timely and targeted treatment and monitor treatment outcomes.

## Supplementary material

Supplementary Table S1 Reported studies on how PROs can become embedded into electronic health records to generate evidence on patient outcomes.
