# Supplementary material for: Leveraging stories of cardiac amyloidosis patients of African ancestry or descent to support patient-derived data elements for efficient diagnosis and treatment
Source: Front Pharmacol. 2023 Nov 24;14:1276396. doi: 10.3389/fphar.2023.1276396 (PMC10704161; doi:10.3389/fphar.2023.1276396)
Supplement: Supplementary file 1 [file Table1.DOCX]

**Supplement**

| **Author (Year)** | **Clinical Practice** | **PRO/PROM Measure or Tool** | **EHR Approach/Capability** | **Treatment Outcomes & Challenges/Limitations** |
| --- | --- | --- | --- | --- |
| Taxter and Natter, 2022 | Pediatric rheumatology | Ability to obtain patient-generated data:   - Questionnaires/patient-reported outcomes - Computer adaptive testing - Home monitoring tools (applications, smart devices) - Graphical depictions of data such as timeline views | Patient Engagement  Patient-portal access | Treatment Outcomes: Not reported.  Challenges/Limitations: Not yet universally implemented, utilization often requires technical sophistication of users |
|  |  | Export and real-time access to clinical data and PROs collected within the EHR | Interoperability: EHR data interfacing with medical apps, registries, and other clinical research  Standardized data elements and  data fields  Fast Healthcare Interoperability  Resources (FHIR) | Treatment Outcomes: Not reported.  Challenges/Limitations: Standard data elements not universally used, FHIR-EHR and FHIR-Registry interfaces in early stages of development or do not yet exist |
| Cella *et al.*, 2023 | Hematology/Oncology | Cancer patient-reported outcomes (cPRO) symptom monitoring and management program that resides within the EHR patient portal (Epic MyChart [MyNM]) and its hyperspace (for in-clinic assessments) - includes Patient Reported Outcomes Measurement Information System® (PROMIS®) measures | Customized EHR-integrated installation implemented across multiple hematology/oncology clinics | Treatment Outcomes: Stepped-wedge, cluster randomized trial will be used to evaluate the impact of implementation strategies (e.g., smart phrases for clinician ease of access to cPRO results, patient-facing education print materials, in-clinic assistance of cPRO) to enhance engagement of patients and adoption by clinicians  Patient outcomes will be evaluated - common cancer symptoms of pain, fatigue, depression, and anxiety, physical function, concerns related to nutrition and practical needs  Challenges/Limitations: Many patients in the health system do not register for the Epic patient-facing portal and will thus not automatically receive cPRO assessments and be reached effectively  MyChart is only available in English, presenting challenges in Hispanic patient enrollment or engagement  MyNM Care Corner does not provide direct communication with clinicians |
